# Thermal challenge and food intake: Mutual regulatory mechanisms

**DOI:** 10.4103/NRR.NRR-D-25-00170

**Published:** 2025-09-29

**Authors:** Alán Alpár

**Affiliations:** 1 Department of Anatomy, Histology and Embryology, Semmelweis University, Budapest, Hungary; 2 SE NAP Research Group of Experimental Neuroanatomy and Developmental Biology, Semmelweis University, Budapest, Hungary

**Keywords:** agouti-related peptide neurons, heat challenge, heat stress, modulation, paraventricular nucleus, proopiomelanocortin neurons, sympathetic nervous system, thermogenesis

## Abstract

Human energy homeostasis principally depends on thermal and food intake regulation mechanisms. In addition to describing the major pathways, brain centers, and their connections which control these functions, this review focuses on (i) interrelations between thermoregulation and food intake, (ii) the role of tanycytes, and (iii) the regenerative capacity of the system in mammals. In the arcuate nucleus, anorexigenic proopiomelanocortin/melanocyte-stimulating hormone neurons and orexigenic neuropeptide Y/agouti-related peptide neurons are reached by a wealth of diet-induced (e.g., leptin, ghrelin, or insulin) or hormonal (e.g., thyroid hormone) signals. These neurons are not only sensitive to temperature signals but also point to both direct and indirect executors of thermoregulation through axonal projection onto the sympathetic system, by producing cleaved proopiomelanocortin products and by reaching endocrine orchestrators, including corticotropin-releasing hormone and thyrotropin-releasing hormone neurons in the paraventricular nucleus. Meanwhile, ambient temperature affects food intake: the warm-cold separated spinobrachial-preoptic area pathways ultimately target agouti-related peptide neurons to attenuate or increase cold-evoked feeding. The brainstem, hypothalamic, and preoptic area centers of these pathways are reached by a wide array of modulatory signals, which refine thermal and dietetic regulation according to conditions such as daily cycle, stress, or fever. We also review regulating mechanisms that are activated in an extreme form of fasting when food becomes unavailable: a specific body temperature control in hibernating animals during the winter months. Understanding this mechanism could likely contribute to medical applications; i.e., artificial induction of a hibernation-like hypometabolic state. Tanycytes uniquely shape energy homeostasis: they dynamically shape the blood–brain barrier to regulate neurohormone sequestration into the brain parenchyme, but also shuttle between the blood circulation and the cerebrospinal fluid bidirectionally. Therefore, blood- or liquor-borne signals not only target arcuate hypothalamic neurons from different circulatory systems, but the activity of diverse groups of neurohormone (such as leptin or ghrelin)-sensitive periventricular neurons will be synchronized. Tanycytes can likely link thermal and food-intake regulations: they carry receptors (most importantly transient receptor potential cation channel subfamily V member 1, glucose, and cytokine receptors) which enable them to sensitively monitor and react to both thermal and energy homeostatic challenges, including anorexia, while they reduce feeding in heat exposure via a specific parabrachial-tanycyte-hypothalamic circuitry. Tanycytes also emerge as the source for the regenerative potential of thermal and energy homeostasis: they not only display functional and morphological plasticity but also neural stem cell properties; they supply the arcuate nucleus with new neurons in both temperature- and diet-responsive manners, which are compromised in extreme/pathological heat challenges or diet-induced obesity, respectively.

## Introduction

Increased environmental heat exposure has merged as a global challenge in recent years. The health impact of climate change is dramatic: a 1°C increase in temperature is positively associated with cardiovascular disease-related mortality across all cardiovascular diagnoses, leading to unexpected death tolls, especially during acute heatwaves (Liu et al., 2022). Neurodegenerative diseases, already among the most burdensome, have been associated with impaired resilience to temperature change, although the link is not fully proven or understood (Sisodiya, 2024). Structured interview-based studies indicated that while elderly people perceive heat strain as challenging, they fail to develop sufficient coping strategies, endangering the health of this most heat-vulnerable risk group (Kemen et al., 2021; Bein, 2024).

The central nervous system of mammals uses various networks to perceive and control temperature changes. The goal of reaction mechanisms is to maintain homeostatic physiological functions. For this, heat-defense responses, which guarantee steady body core temperature (Notley et al., 2023), do not suffice alone; mechanisms that shape water and food intake, energy expenditure, physical activity, and corresponding behavior are required, which may further include hormone mirror adjustment (Rogers et al., 2024). Similarly, cold exposure triggers a coordinated response through metabolic, endocrine, autonomic, and behavioral systems (Machado and Saper, 2022).

In this review, we focus on how the brain reacts to thermal challenge. We first describe the pathways that convey thermal signals towards and within the central nervous system. We then describe the location, cell types, and connectivity of the brain thermal center responsible for shaping adequate responses to maintain homeostasis. Major cellular mechanisms, receptors, and pathways behind functions are also highlighted to identify future – or present – pharmatherapeutic targets. Also summarized are the canonical regulatory circuitry and mechanism, as well as several modulatory pathways of different functional systems which shape thermogenesis, including blood-borne-signals like leptin or insulin, stress signaling receptors (signaling via adrenergic or corticotropin-releasing factor), or prostaglandins (see appropriate chapters). The review especially concentrates on mechanisms that mutually link thermal challenges to food intake; in this section, we summarize the brain centers and the cell types, as well as their cellular mechanisms and connectivity through which they emerge as key players in this vital homeostatic system. Finally, we investigate those neuroanatomical features that propose the regenerative potential of food intake and thermoregulatory mechanisms.

## Search Strategy

Studies mentioned in this narrative review, published between database inception and May 2025, were searched on the PubMed database using these keywords: agouti-related peptide neurons, heat challenge, heat stress, modulation, paraventricular nucleus, proopiomelanocortin neurons, sympathetic nervous system, thermogenesis.

## Sensing the Temperature – Pathways, a Brain Center, Their Connectivity and Interactions

The core temperature of the body can shift due to internal (e.g., fever or physical exercise) or environmental causes. In both cases, neuronal elements ensure the sensing of temperature. It is typically acknowledged that skin and visceral thermoreceptors (Leva and Whitmire, 2023) monitor outer and inner temperatures, respectively; however, select neuron pools in the brain have developed the remarkable ability to sensitively react upon brain thermal change itself. Thus, in addition to the canonical pathway in which well-characterized TRPM8 cold (Peier et al., 2002; Huffer et al., 2024) and transient receptor potential cation channel subfamily V member 1 (TRPV1) warm receptors (Yarmolinsky et al., 2016; Abd El Hay et al., 2025) mediate typically innocuous thermal signals through the spinothalamic tract towards the primary somatosensory cortex, the hypothalamus harbors neurons in its preoptic area (PoA) which are highly reactive to temperature changes per se (Lewis, 2025). Approximately 30% of the neurons of this tiny area between the optic chiasm and the anterior commissure play a vast and multifaceted role in thermal regulation. Its anatomical position – proximate and directly connected to hypothalamic homeostasis and behavioral centers – makes it a powerful regulator of thermal balance (Morrison and Nakamura, 2019; Nakamura et al., 2022a); moreover, their experimental inducibility in primates, likely offering future therapeutic potential, has been successfully addressed recently (Zhang et al., 2023). Their ability to sense thermal challenge enables them to respond to healthy and illness-related temperature changes, such as physical exercise or fever, respectively, which can increase brain temperature by 2–3 °C (Fuller et al., 1998).

**Figure 1:**
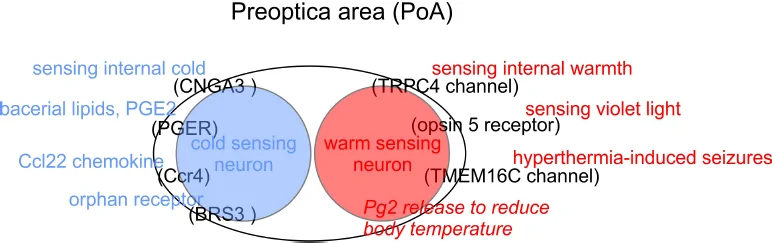
Channels and receptors in temperature-sensitive neurons of the preoptic area. PoA neurons express a wide array of receptors. This figure gives a simplified model of channels and receptors which enable them to react to different kinds of stimuli. Their activation reduces or increases body temperature in warm- and cold-sensing neurons, respectively. Specific stimuli that activate the channels are inside parentheses. BRS3: Bombesin receptor subtype 3; Ccl22: C–C motif chemokine ligand 22; Ccr4: C–C chemokine receptor type 4; CNGA3: cyclic nucleotide-gated cation channel alpha-3; PGD2: prostaglandin D2; PGE2: prostaglandin E2; PGER: prostaglandin E receptors; TMEM16C: transmembrane member 16C voltage-gated calcium-activated anion channel; TRPC4: transient receptor potential cation channel subfamily C member 4.

While the cellular and molecular mechanisms by which PoA neurons measure temperature have remained largely undefined, important advances have been made (**Figure 1**). Thus, the TRPC4 channel was identified as being essential for sensing internal warmth: TRPC4 antagonists and agonists bidirectionally regulated core temperature and TRPC4 channel silencing in mice with substantially attenuated hypothermia, induced by light-mediated heating of the PoA (Zhou et al., 2023). A genome-wide association study identified TMEM16C, a membrane protein that facilitates sodium-activated potassium currents, as a risk factor for febrile seizure; accordingly, its experimental deletion from PoA neurons led to a deficiency in thermoregulation and greater susceptibility to hyperthermia-induced seizures (Wang et al., 2021). Surprisingly, a subset of warm-sensitive PoA neurons carries opsin 5 receptors, which enable them to act as a violet light-sensitive deep brain photosensitive unit that suppresses BAT thermogenesis (Zhang et al., 2020). Single-cell RNA-sequencing combined with whole-cell patch-clamp recordings and chemogenetics identified the *Ptgds* gene – encoding the enzyme that synthesizes prostaglandin D2 – as a genetic marker for temperature-sensitive PoA neurons (Wang et al., 2019). Rising temperature triggers prostaglandin D2 release, which excites downstream neurons in the ventral part of the preoptic area that mediates body temperature decrease (Wang et al., 2019). Of note, *Ptgds* is not uniquely restricted to temperature-sensitive neurons; besides its non-neuronal expression and functions, it is expressed in various brain regions to shape, amongst others, conditioned fear memory in the hippocampus (Shen et al., 2023) or optic nerve damage-induced inflammation in the visual cortex (Li et al., 2025a).

All the above data show that PoA neurons have been typically characterized to reduce body temperature when activated; in contrast, activation of bombesin-like receptor 3 (POABRS3) –carrying PoA neurons increases body temperature (Pinol et al., 2021). For cold-sensing neurons, p38α-triggered downstream pathways were implicated in reducing energy expenditure – but not extracellular signal-regulated kinase 1/2 and c-Jun N-terminal kinase (Wang et al., 2022). Interspecies differences have been identified between mice and hibernating squirrels: unlike squirrels, mice express the cyclic nucleotide-gated ion channel CNGA3, which is potentiated in low temperatures; hence, it acts as a cold sensor and drives downstream thermoregulation (Feketa et al., 2020). In addition, PoA neurons can also be involved in fever, a regulated form of hyperthermia, when the adaptive reference mechanism (“set-point”) of body temperature is shifted to a higher value and actively maintained (Nakamura, 2024). Thus, PoA neurons can carry receptors, which make them triggerable by pyrogens, such as prostaglandin E2, whose production is upregulated in fever by bacterial lipids (Osaka, 2022). A similar example is C–C chemokine receptor type 4, which binds C–C motif chemokine ligand 22 induced fever via activation of the brown adipose tissue; this could be reduced by indomethacin pre-treatment, suggesting the involvement of cyclooxygenase activation; hence, the formation and action of the pyrogen prostaglandin E2 (Osborn et al., 2011).

**Figure 2:**
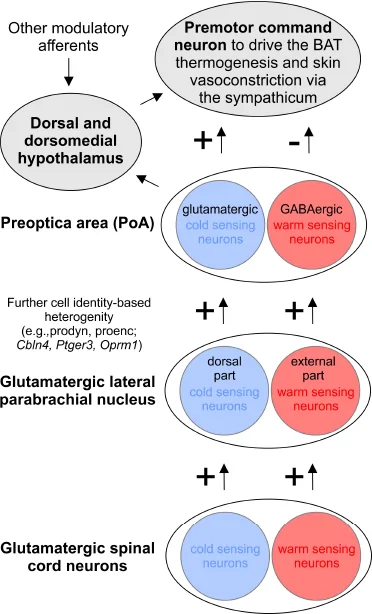
Canonical flow of thermal information in the central nervous system for thermal regulation. Warm- and cold-sensing neurons throughout the spinal cord, parabrachial nucleus of the brain stem, and the preoptic area form parallel pathways with additional cell heterogeneity and alternative routes.

In addition to their innate thermoreceptive ability, PoA neurons are wired into a circuitry through which they (i) receive signals from the periphery; i.e., from viscera and skin, which carry feedback and feedforward signals, respectively, and (ii) activate the sympathetic nervous system after signal integration (McKinley et al., 2021; **Figure 2**). Thus, in the afferent limb, information from spinal cord funicular neurons arrives through the lateral parabrachial nucleus into the PoA, whereas efferents relay through the dorsal and dorsomedial hypothalamus to reach premotor command neurons mainly in the rostral raphe pallidus nucleus to drive the preganglionic sympathetic neuron in the intermediolateral nucleus of the spinal cord (Nakamura et al., 2022a). Thermosensory transmission through the spinoparabrachial pathway is essential for PoA neurons: defunct lateral parabrachial nucleus function leaves rats unprotected towards warm and cold, and the animals cannot maintain their body core temperature (Nakamura and Morrison, 2010). Of note, the parabrachial nucleus harbors a specific subset of neurons, which transmit real or potential threat signals; these CGRP-containing cells convey thermal or visceral noxious signals towards the extended amygdala (Palmiter, 2018). Thermoresponsive spinoparabrachial neurons are glutamatergic, subdivide into innocuous warm and cold sensing neurons, which relay warm and cold through the dorsal and external part of the nucleus, respectively, and define separate neuron populations in the PoA, too (Nakamura and Morrison, 2010). Cold activates glutamatergic PoA neurons, whereas warmth activates GABAergic PoA neurons, which excite or inhibit the sympathetic premotor command neurons to increase or reduce, respectively, thermogenesis; i.e., skin vasoconstriction and brown adipose tissue thermogenesis. Of note, other sympathetic premotor command neurons in the rostral ventrolateral medulla drive an opposite mechanism; namely, sweating, which increases heat loss (McAllen and McKinley, 2018). The specific circuitry that connects the PoA and RVMM is, nevertheless, unknown. Alternatively, glutamatergic and GABAergic PoA efferents relay through the dorsomedial hypothalamus nucleus/dorsal hypothalamic area, which allows additional regulatory mechanisms, including a disinhibition (i.e., GABAergic PoA neurons inhibit GABAergic hypothalamic neurons) pathway and the reception of other modulatory afferents (Nakamura et al., 2022b). Despite extensive tracing studies and a recent robust single-cell RNA-sequencing-based study using 21 Cre-driver lines of mice (Pauli et al., 2022), the clinical relevance of heterogeneity in the parabrachial–PoA axis is still not understood. The druggability of the opioid system makes prodynorphin-proenkephalin-expressing PoA-projecting parabrachial neurons especially promising: photostimulation in corresponding Cre-mouse lines showed that these glutamatergic warm-activated cells generated a rapid onset of hypothermia, caused vasodilation and suppressed brown fat thermogenesis, and drove thermal defensive behaviors (Norris et al., 2021).

## Modulatory Pathways of Different Functional Systems Target PoA Neurons to Shape Thermogenesis

**Figure 3:**
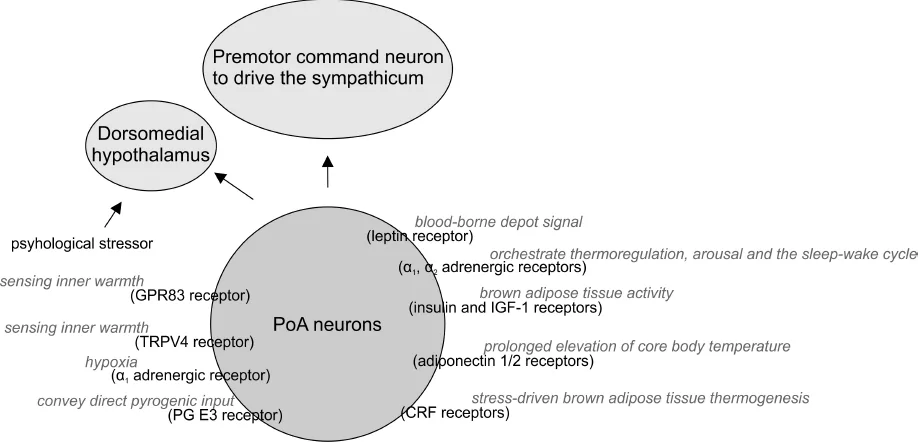
Modulators of preoptic area neurons in thermal regulation. Major receptor types which convey distinct information for preoptic area neurons. Specific receptors which mediate the effects are inside parentheses. CRF: Corticotropin-releasing factor; GPR83: G-protein coupled receptor 83; IGF: insulin-like growth factor; PG: prostaglandin; TRPV4: transient receptor potential cation channel subfamily V member 4.

Although the discharge of sympathetic premotor neurons principally depends on the balance of the glutamatergic/GABAergic PoA-dorsal hypothalamus interplay, a wealth of modulatory afferents modulates thermogenesis effectively (**Figure 3**). These include blood-borne signals, such as the neurohormone leptin, which reflect principal information about the status of the long-term energy depot of our body (Takahashi et al., 2024). Many PoA and dorsomedial hypothalamic neurons carry leptin receptors (Francois et al., 2025), and leptin-triggered thermogenesis can be blocked by 5-HT1A receptor activation in the raphe pallidus (Morrison, 2004). Of note, many leptin-responsive PoA neurons co-express alpha(1A)- and/or alpha(2A)-adrenergic receptor (AR) isoforms, which likely enable the pretectal area to orchestrate thermoregulation, arousal, and the sleep-wake cycle (Frontini and Giordano, 2010; Nakamura, 2024; Mondino et al., 2025). Insulin receptors and insulin-like growth factor 1 (IGF-1) receptors also decorate PoA neurons, which turn insulin and IGF-1 signals into brown adipose tissue activity (Sanchez-Alavez et al., 2011; Barrios et al., 2021). Warm sensitive PoA neurons can carry adiponectin type 1 and type 2 receptors (AdipoR1 and AdipoR2) (Klein et al., 2011), and local administration of adiponectin has been shown to induce prolonged elevation of core body temperature and decreased respiratory exchange ratio, whereas hyperthermic effects were blunted in both AdipoR1 deficient and AdipoR2 deficient mice (Klein et al., 2011). Further, PoA neurons can carry corticotropin-releasing factor receptors, which allow a stress-driven brown adipose tissue thermogenesis through the dorsomedial hypothalamus-medullary raphe pallidus pathway (Cerri and Morrison, 2006). Psychological stress-induced hyperthermia finds an alternative route: *in vivo* drug nanoinjections followed by thermotelemetry showed that the dorsomedial hypothalamus functions as a hub for stress signaling, monosynaptically projecting both to the rostral medulla and to the paraventricular nucleus to shape sympathetic and endocrine output, respectively (Kataoka et al., 2014). Sleep and daily cycle also affect thermogenesis (Rothhaas and Chung, 2021), which is transferred by an orexinergic pathway from the perifornical nucleus to the raphe pallidus (Tupone et al., 2011). Further mechanisms also contribute to thermogenesis in sleep: ventrolateral galanin-expressing PoA neurons couple sleep induction and heat loss, with opto- and chemogenetic activation of these neurons promoting sleep and reduced body temperature (Kroeger et al., 2018). In a state of illness, specific mechanisms evolved: GABAergic warm sensitive neurons carry prostaglandin EP3 receptors to convey direct pyrogenic input towards the dorsomedial hypothalamus (Nakamura et al., 2005). Further receptors, with unidentified/incompletely identified downstreams were shown to regulate thermogenesis in PoA neurons, including transient receptor potential cation channel subfamily V member 4 (TRPV4) (Yadav et al., 2017) or GPR83 receptors (Dubins et al., 2012), α1-adrenoceptors, and nitric oxide synthase in hypoxia (Osaka, 2011). Of note, local ethanol administration increases PoA neuronal activity but does not influence ethanol-induced changes in core body temperature (Westerman et al., 2010).

Notably, the preoptic area shows regenerative potential. Constant exposure to moderate heat facilitates progenitor cell proliferation and neuronal differentiation in the hypothalamus (Matsuzaki et al., 2017). The neural phenotype and heat responsiveness of hypothalamic newborn cells is remarkable: both warm-sensing glutamatergic and cold-sensing GABAergic PoA neurons are generated and can indeed be activated by high ambient temperature (Matsuzaki et al., 2017). These newly born heat responsive neurons play a critical role in long-term heat acclimatization (Matsuzaki et al., 2017).

## Food Intake Shapes Thermal Homeostasis

**Figure 4:**
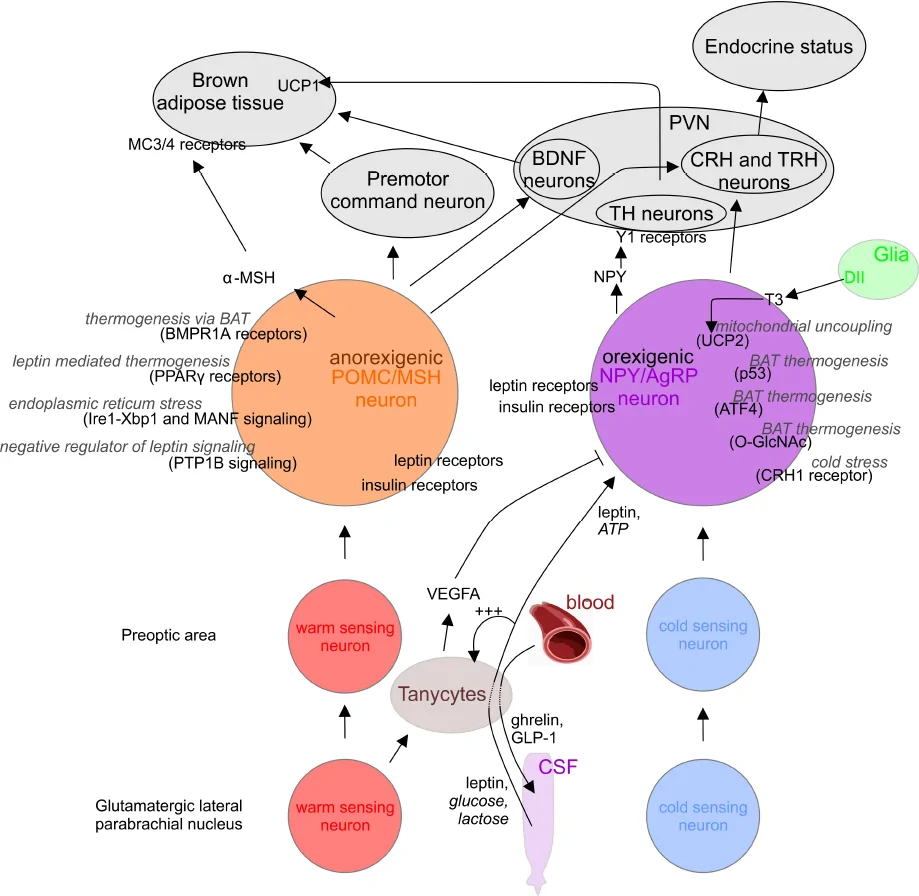
Interactions between the brain centers of thermoregulation and food intake. Warm- and cold-sensitive preoptic neurons contact POMC and AgRP neurons of arcuate nucleus, respectively. Orexinergic POMC and anorexinergic AgRP neurons receive input through different signaling pathways which ultimately shape thermogenesis. Arcuate neurons contribute to thermal control through the hypothalamic paraventricular nucleus and the sympathetic nervous system. Tanycytes shuttle information about food and thermal homeostasis bidirectionally between the blood stream and the cerebrospinal fluid. For details, see the main text body of the manuscript. Specific receptors/pathways/molecules which mediate the effects in POMC/MSH or NPY/AgRP neurons are inside parentheses. AFT4: Activating transcription factor 4; AgRP: agouti-related peptide; BDNF: brain-derived neurotrophic factor; BMPR1A: bone morphogenetic protein receptor, type IA; CRH: corticotropin-releasing hormone; CSF: cerebrospinal fluid; GLP-1: glucagon-like peptide 1; Ire1-Xbp1: inositol-requiring enzyme 1/X-box binding protein 1; MANF: mesencephalic astrocyte-derived neurotrophic factor; MC3/4: melanocortin-3 and -4 receptors; MSH: melanocyte-stimulating hormone; NPY: neuropeptide Y; O-GlcNAc: O-linked β-N-acetylglucosamine; p53: p53 tumor suppressor gene; POMC: proopiomelanocortin; PPARγ: peroxisome proliferator-activated receptor gamma; PTP1B: protein tyrosine phosphatase 1B; PVN: paraventricular nucleus of the hypothalamus; TH: tyrosine hydroxylase; TRH: thyrotropin-releasing hormone; UCP 1 and 2: mitochondrial uncoupling proteins 1 and 2; VEGFA: vascular endothelial growth factor A.

Human thermal homeostasis and food intake homeostasis are mutually and bidirectionally linked to, and shaped by one another (**Figure 4**). Nevertheless, most data underline a food intake/fasting → thermogenesis/thermal loss action pathway. Beyond obligatory thermogenesis as a response to daily body function, facultative thermogenesis reflects an adaptive increase in energy expenditure (Saito et al., 2020), which clearly correlates with food intake. Neurons responsible for this regulation were identified in the arcuate nucleus of the hypothalamus, a diencephalic area closely related to the median eminence where the blood–brain barrier is missing (Pfau et al., 2024). In this area, anorexigenic proopiomelanocortin/melanocyte-stimulating hormone (POMC/MSH) neurons and orexigenic neuropeptide Y/agouti-related peptide (NPY/AgRP) neurons establish a delicate balance for food intake mechanisms (Ribeiro et al., 2025), but the downstream of these neurons points to both direct and indirect executors of thermoregulation. POMC/MSH neurons are wired, via the solitary tract nucleus-rostral raphe pallidal neuron chain, onto spinal cord intermediolateral neurons to activate the sympathetic nervous system, which, in addition to its pilo-, sudo- and vasomotor innervation in the skin, also shapes brown adipose tissue thermogenesis (Tan and Knight, 2018). Further, the cleaved product of POMC, alpha-MSH, itself activates melanocortin 3 and 4 receptors to induce thermogenesis in the brown adipose tissue (Chitravanshi et al., 2016). Oppositely, NPY/AgRP neurons inhibit thermogenesis via a direct Y1 receptor-mediated tyrosine hydroxylase signal regulation in the paraventricular hypothalamic nucleus, which is also associated with reduced tyrosine hydroxylase expression in catecholaminergic brain stem nuclei, including the locus coeruleus (Shi et al., 2013). At the same time, POMC/MSH and NPY/AgRP neurons reach endocrine orchestrator neurons in the paraventricular nucleus; this hypothalamic peptidergic circuitry allows arcuate neurons to directly shape corticotropin-releasing hormone and thyrotropin-releasing hormone neuron activity, and thus, endocrine thermal status (Berthoud, 2002).

If efferent signals from arcuate neurons shape thermal homeostasis, afferent signals to these neurons must be powerful regulators of thermogenesis or thermal loss. Food intake, or the lack of it, typically creates such signals, which reflect the present or predicted energy homeostasis of the body. In diet-induced thermal control, leptin and insulin emerge as major signals, and arcuate neurons carry leptin and insulin receptors to transfer ligand binding into a thermal effect (Tran et al., 2022). Leptin decreases appetite and increases sympathetic nerve activity, which elevates energy expenditure in the brown adipose tissue (Wang et al., 2020). This mechanism is robustly exemplified by a leptin-induced brain-derived neurotrophic factor (BDNF) pathway regulating sympathetic innervation of adipose tissue: POMC neurons activate BDNF-expressing hypothalamic paraventricular neurons upon leptin binding, shaping a top-down neural pathway for energy homeostasis (Wang et al., 2020). Several further ligands trigger POMC neuron-regulated thermogenesis, including central bone morphogenetic protein, which binds onto POMC neurons to drive bone morphogenetic protein receptor, type IA-mediated thermogenesis in the brown adipose tissue via activation of the sympathetic nervous system (Townsend et al., 2017).

Several cellular mechanisms were identified when altered sensitivity of POMC neurons shaped specific aspects of thermal regulation. Peroxisome proliferator-activated receptor γ coactivator-1β containing POMC neurons elevated body core temperature through mediating the effect of leptin on thermoregulation in mice; nevertheless, it was only observed in the fed state (Delezie et al., 2020). Endoplasmic reticulum stress-triggered dysregulation of body temperature was shown in inositol-requiring enzyme 1/X-box binding protein 1 (Ire1-Xbp1) mediated and mesencephalic astrocyte-derived neurotrophic factor mediated signaling: their selective deletion in POMC neurons predisposed those neurons to leptin and insulin resistance, which caused inability to induce thermogenesis on a high-fat diet, finally leading to obesity (Tang et al., 2022). Leptin activation of POMC neurons increases thermogenesis and locomotor activity; as for the inner cellular mechanism, the homeostatic response to cold exposure through leptin signaling was shown to be mediated by protein tyrosine phosphatase 1B, an important negative regulator of leptin signaling (De Jonghe et al., 2011).

Yet not only anorexigenic POMC/MSH neurons but also orexigenic NPY/AgRP neurons are reached by peripheral signals, which shape thermoregulation. AgRP neurons are activated by both thermal and dietetic signals; cold activates AgRP neurons, which increase food intake in mice (Deem et al., 2020) through a PoA-AgRP neuronal pathway (Yang et al., 2021), but *in vivo* fiber photometry and indirect calorimetry showed that a high-fat diet can disrupt the functioning of this thermoregulatory system (Deem et al., 2023). Cold can also induce thermogenesis by stress signaling: a subset of AgRP neurons in the hypothalamic arcuate nucleus carries corticotropin-releasing factor receptor type 1, which allows these neurons to modulate sympathetic nervous system activity to adapt to cold stress via thermogenesis, at least in mice (Kuperman et al., 2016). Thermogenesis of brown adipose tissue principally shapes diet induced obesity. p53, a tumor suppressor, emerged as an important molecular regulator in this process: p53 deficiency or overexpression in AgRP neurons caused reduced brown adipose tissue and diet-induced obesity or decreased body weight and stimulated brown adipose tissue thermogenesis, respectively, which was mediated via the c-Jun N-terminal kinase pathway (Quinones et al., 2018). Similarly, the lack of the transcription factor 4 in AgRP neurons made mice resistant to high-fat diet-induced obesity, reduced food intake and enhanced thermogenesis, in this case in the brown adipose tissue (Deng et al., 2017). A specific aspect of diet-induced thermogenesis occurs through the control of beige adipocytes, which causes the browning of white fat and improves energy metabolism. Feeding reduces the phosphatase TCPTP in the hypothalamus, which increases AgRP/NPY neuronal insulin signaling, a mechanism, which is defective in obesity: mice lacking TCPTP in AgRP/NPY neurons were resistant to diet-induced obesity and increased beige fat activity and energy expenditure (Dodd et al., 2017). An alternative mechanism in fasting is mediated by O-GlcNAc transferase in AgRP neurons: food intake restriction elevates its level, which suppresses thermogenesis to conserve energy in response to fasting (Ruan et al., 2014). The activity of AgRP neurons is further shaped by local thyroid hormone synthesis and a non-neuronal effect: fasting increases the expression of the type 2 deiodinase enzyme in glial cells, which contact AgRP neurons (Coppola et al., 2007). The increased active thyroid hormone level is linked with mitochondrial uncoupling through a mitochondrial uncoupling protein 2-dependent mechanism (Coppola et al., 2007).

## When Temperature Shapes Food Intake

In contrast to the wealth of identified mechanisms for how food intake shapes thermal regulation, less is known about the opposite mechanism, although simple empirical evidence suggests that heat or cold exposure reduces or enhances food intake, respectively. A pilot randomized controlled trial showed that warm ambient temperature decreased food intake in a simulated office setting (Bernhard et al., 2015). The question of how ambient temperature affects food intake has gained new momentum due to climate change, dietetic reasons, engineered sport activity, and specific free-time activities (e.g., sauna). The warm-cold separation within the spinobrachial-PoA pathway has been further traced in the arcuate nucleus: mPoA neurons project to AgRP neurons to evoke eating behavior in cold (Yang et al., 2021). Thus, cold stimulation triggers an immediate response pattern in calcium signals, and inhibition or activation of either AgRP neurons or arcuate nucleus-projecting mPoA neurons attenuates or increases cold-evoked feeding, respectively (Yang et al., 2021). 

A non-canonical feeding center of the preoptic area, namely its anteroventral and periventricular portions (apMPoA), can respond to altered dietary states. In this system, parallel pathways emerge in a parabrachial nucleus – apMPoA – arcuate or paraventricular hypothalamic nuclei neuron chain. Thus, afferents from the external and dorsal subnuclei of the lateral parabrachial nucleus project to neighboring but distinct neuronal populations within the apMPoA, which sense low temperature and promote food intake (arcuate nucleus projecting neurons) or sense high temperature and suppress food intake (paraventricular nucleus projecting neurons), respectively (Qian et al., 2022). The dependence of energy balance upon body temperature is not mandatory: PoA neurons upon leptin binding modulate energy expenditure in response to internal energy state instead (Yu et al., 2018). In other words, PoA neurons should not be considered only as body temperature regulators, but also as neurons, which shape body weight control. Leptin receptor-carrying glutamatergic, but not GABAergic, PoA neurons activated by viral vectors in mice drove robust suppression of energy expenditure and food intake, which lowered body temperature and body weight (Yu et al., 2016).

Recently, virus-mediated gene manipulation and circuit mapping combined with electrophysiology, optogenetics, mass spectrometry, and behavioral testing unveiled a novel brainstem-hypothalamus neuronal circuit, which reduces feeding upon heat exposure (Benevento et al., 2024). In this mechanism, parabrachial thermosensing glutamatergic neurons activate tanycytes either directly or through second-order hypothalamic neurons. Although tanycytes are typically known for secreting into the cerebrospinal fluid, in this neuronal circuit a parabrachial nucleus – tanycyte – arcuate nucleus information flow emerged (Benevento et al., 2024). Thus, the tanycyte-produced vascular endothelial growth factor A is not discharged into the ventricular space but is released along the dendrites of the tanycytes into the parenchyme of the arcuate nucleus. Here, vesicle-associated membrane protein 2-dependent exocytosis of vascular endothelial growth factor A increased the spike threshold of *Flt1*-expressing dopamine/AGRP neurons to prime an anorexigenic output.

Nevertheless, a change in ambient temperature does not necessarily ask for a dietetic change in the animal kingdom. An extreme form of fasting occurs in hibernating animals when food intake reduces dramatically during the winter months. Certain hibernator species undergo complete aphagia for over half a year, irrespective of ambient temperature. The evolutionary sense of this mechanism is to completely cease feeding and robustly decrease body temperature in order to survive, i.e., conserve energy, when food is virtually unavailable and ambient temperature is low (Mota and Madden, 2024). Here, a different mechanism in the temperature-diet relation is presented: instead of a short-term rhythm, which shapes energy expenditure according to body/ambient temperature, an endogenous “sliding adaptive reference mechanism” is established and maintained, which sets a different “ideal” body mass for each season. Although the role of the hypothalamus is mandatory (Florant and Healy, 2012), seasonality overwrites temperature-dependent food-intake regulatory mechanisms in non-hibernating mammals. Thus, despite the fact that (i) non-hibernating rodents and hibernators share similar food intake pathways within the brain with their arcuate nucleus containing similar NPY/AGRP and POMC/CART neuron populations and (ii) the arcuate nucleus is sensitive to circulating neurohormones (Florant and Healy, 2012), the rhythm and the outcome of these impulses are completely different. Independent from ambient temperature, it is the increase of free fatty acids, leptin, and insulin, due to the massively accumulated body fat during the summer months, which introduces torpor, but only coincides with oncoming low temperature during the winter months (Florant et al., 1990). Daurian ground squirrels (*Spermophilus dauricus*) that were exposed to cold for 3 weeks in late summer increased hypothalamic orexigenic neuropeptides but not food intake, suggesting that the fattening process is principally controlled by a strong circannual rhythm (Xing et al., 2020).

Moreover, the ability to actively lower the body temperature to reduce energy expenditure, including food intake, is seemingly not an exclusive characteristic of hibernating animals. In this process, similarly to hibernating animals, the body temperature decreases because of a regulated shift in the adaptive reference mechanism (“set-point”). Recently, a discrete neuronal circuit located in the hypothalamus was shown to trigger and regulate a hibernation-like state, namely, anapyrexia, in rodents. Short-term hypometabolic states known as daily torpor do occur even in lab mice when the sensitivity of the thermoregulatory system is reduced (Sunagawa and Takahashi, 2016). Many medical applications would benefit from an artificial induction of a hibernation-like hypometabolic state (Uchino et al., 2024) in mammals, including humans. Based on bioinformatics and reverse pharmacology data, a neuropeptide, pyroglutamylated RFamide peptide (QRFP), emerged as a key player in this process. QRFP was exclusively expressed by hypothalamic neurons, and chemo- or photogenetic activation of such neurons in the medial preoptic area in a corresponding transgenic strain (*Qrfpi*^*Cre*^ mice) led to hypothermia and immobility (Takahashi et al., 2020). This Q-neuron-induced hypothermia and reduced food intake were not due to organ or tissue damage, and no such harm was evoked by this state (Takahashi et al., 2020). Q-neurons of the medial preoptic area effectively shaped thermogenesis through a dorsomedial hypothalamus-raphe pallidus neuron chain, hence via activating the sympathetic limb. Of note, bodily functions and behavior are still regulated to adapt to a change in ambient temperature in this specific hypothermia-like state (Takahashi et al., 2020). While the physiological role of this unique neuronal subset in the hypothalamus remains currently unknown, they likely offer an exciting possibility in thermal and food-intake regulation since they stabilize circadian fluctuation in body temperature and might sense humoral factors that are released by tanycytes and ependymal cells through their dendrites extending along the circumventricular space (Takahashi et al., 2020). Of note, Q-neurons represent a subpopulation of warm-sensing preoptic neurons that co-express pituitary adenylate cyclase-activating polypeptide and BDNF (Tan et al., 2016).

In mammals, low ambient temperature typically increases brown adipose tissue thermogenesis, which can simultaneously activate food-seeking behavior. One candidate cell group/molecule, which can co-trigger the sympathetic nervous system to activate brown adipocytes with food-seeking activity pathways is orexin. Orexin is an orexigenic peptide (Lee et al., 2021); together with its co-released transmitters, orexin neurons enhance brown adipose tissue thermogenesis, and the ablation of these neurons leads to lower BAT activity and *Ucp1* expression in response to stress (Zhang et al., 2010). At the same time, orexin itself promotes food-seeking behavior and has an important role in arousal, locomotor activity, and reward (Straat et al., 2020). This is likely to be advantageous during phylogenesis (Straat et al., 2020).

An important interrelation between temperature and food intake emerges under pathological conditions. Fever induced by peripheral inflammation couples with lethargy and anorexia, and these are mediated through different reaction pathways. Whether fever and anorexia are independent phenomena or whether it is fever, which induces anorexia remains unaddressed in most studies. Ciliary neurotrophic factor (CNTF), leptin, and lipopolysaccharide are effective pyrogens and decrease food intake by inducing cytokines acting on cytokine receptors. In sickness-like conditions characterized by fever and anorexia, the levels of interleukin (IL-6) and tumor necrosis factor-α, as well as relevant inflammatory transcription (such as STAT-3 or nuclear factor kappa B [NF-κB]) increase in the hypothalamus, although a causative link between hyperthermia and reduction of food intake remains ambiguous (Dangarembizi et al., 2018). Similarly, prostaglandins are critical signaling elements of immune system challenges, which typically cause fever and anorexia. These effects, nevertheless, are mediated by anatomically distributed sites rather than a single center, and only intracerebroventricular or paraventricular PGE₂ delivery causes fever and anorexia simultaneously, suggesting a causative link between the two effects in this specific locus of hypothalamus (Skibicka et al., 2011). Indeed, interleukin-1 (IL-1) induces Fos expression in both pro-opiomelanocortin- and neuropeptide Y-expressing neurons in and around the arcuate nucleus, and it is the net output of this cell group, which serves normally to restrain cytokine-induced reductions in food intake IL-1-induced anorexia (Reyes and Sawchenko, 2002). Lately, a direct link between fever and anorexia emerged, based on the presence of TRPV-1-like receptors of POMC neurons in the arcuate nucleus of the hypothalamus. Electrophysiology and behavioral analysis in relevant knock-out and Cre- knock-in in mice showed that acute elevations in hypothalamic temperature triggers acute reductions in food intake via a melanocortinergic circuit: Hyperthermia, induced by treadmill running, reduced food intake, and this was shown to be reversible by selectively inhibiting the *TRPV1* gene in arcuate POMC neurons (Jeong et al., 2018).

## Role of Tanycytes in Thermal Regulation and Food Intake

In general, tanycytes around the third ventricle are typically known to form a physical barrier towards the ventricular space, thereby sequestering neurohormones to the parenchyme of the hypothalamic circumventricular space (Prevot et al., 2018; Gomez et al., 2024; Groh et al., 2024). The system shows considerable functional plasticity. In the median eminence, while fasting or feeding widens or narrows the blood-brain barrier in the parenchyme, respectively, tanycytes strengthen or weaken this blockade during this process to amplify sequestration (Langlet et al., 2013; Brunner et al., 2024). In the arcuate nucleus, tanycytes dynamically tune the blood-brain barrier to regulate the level of circulating metabolites and neurohormones (such as glucose and ghrelin, respectively) upon food intake or fasting (Langlet et al., 2013). However, tanycytes react to nutritional status not only through the modulation of capillary fenestration (Prevot et al., 2021). Due to their unique localization, i.e., with their processes spanning from parenchymal vessels to the ventricular wall, they shuttle bidirectionally between the blood circulation and the cerebrospinal fluid (CSF). Thus, tanycytes were traced to convey blood-borne molecules to the CSF in the following way: (i) leptin from the blood was fluorescently labeled (ii) these molecules exit the vasculature in the arcuate nucleus, (iii) molecules are internalized by tanycytes, (iv) molecules are transported intracellularly and released into the CSF, which finally results in (v) synchronous activation of a diverse group of leptin-sensitive periventricular neurons (Faouzi et al., 2007). A similar conduit applies for other blood-borne metabolites, which regulate food intake: ghrelin (Cabral et al., 2014) and glucagon-like peptide-1 (GLP-1) (Secher et al., 2014) are effectively transported from the arcuate nucleus towards the ventricular space. GLP-1 pharmacological analogues used in type 2 diabetes mellitus and overweight treatment (Dong et al., 2021; Bombassaro et al., 2024) are likely shuttled via tanycytes (Halawi et al., 2017; Imbernon et al., 2022). Furthermore, intracerebroventricular administration of leptin immediately enhances pSTAT3 expression in tanycytes, activating arcuate neurons within minutes via leptin receptors (Faouzi et al., 2007). Blockade of vesicle-associated membrane proteins 1–3 disables leptin transport through tanycytes into the mediobasal hypothalamus, which leads to increased food intake and abdominal fat deposition (Duquenne et al., 2024).

An intriguing aspect of tanycyte function in food-intake mechanism is reflected in their enzymatic machinery, which enables them to act as glucose-sensors (Salgado et al., 2021): similar to pancreatic β-cells, they express the glucose transporter 2, glucokinase (the enzyme, which phosphorylates internalized glucose), and adenosine triphosphate-inhibited potassium channels (K_ATP_). While arcuate NPY/AgRP and POMC neurons regulate energy balance by being electrically inhibited or excited by extracellular glucose, respectively (Fioramonti et al., 2007), tanycytes detect glucose level changes in the CSF and release paracrine factors, including ATP, to activate arcuate neurons (Frayling et al., 2011). *Ex vivo* testing showed that glucose administration dramatically increased ATP release via the hemichannel connexin 43, which led to the paracrine activation of neighboring tanycytes via P_2_Y_1_ purinergic receptors (Frayling et al., 2011). Of note, a similar cellular mechanism can be triggered via the Tas1r2, a sweet taste receptor commonly known to occur throughout the gastrointestinal system, including the tongue (Benford et al., 2017). This amplification mechanism of tanycyte recruitment to release ATP (or lactate) upon glucose level changes in the CSF is an alternative information source to fine-tune NPY/AgRP and POMC neurons in the arcuate nucleus, which primarily receive information about glucose supply from the blood. A specific form of tanycyte function and morphology was found in hibernating animals: in the Arctic ground squirrel (*Urocitellus parryii*) tanycytes, but not astrocytes, adenosine kinase activity decreased during the winter. Since the onset of hibernation critically depends on A1 adenosine receptor (A_1_AR) signaling, tanycytes likely contribute to this specific, seasonal form of robust food-intake reduction (Frare and Drew, 2021).

Tanycytes, further, are also involved in an extreme form of food-intake pathology, anorexia. Chronic diseases trigger systematic inflammation via mediators, with the prototypical member being interleukin-1 beta (IL-1β). IL-1β directly stimulates NF-κB activity in tanycytes as shown in NF-κB-enhanced green fluorescent protein reporter mice in vivo, and induced the mRNA levels of the immediate-early gene Ptgs2, encoding the proinflammatory protein cyclooxygenase-2 (Cox-2), at least *in vitro* (Bottcher et al., 2020). This happens through the canonical signaling pathway that crucially depends on NEMO. Of note, IL-1β induced anorexia depended on tanycytic NEMO, and fever and lethargy were not affected by glial *Nemo* knockout. The release of the anorexigenic prostaglandin E2 from tanycytes can modulate orexigenic and anorexigenic peptides in the hypothalamus, with tanycyte-specific deletion of *Nemo* being sufficient to interfere with IL-1b-induced anorexia. Therefore, tanycytes emerge as an attractive target to ameliorate cachexia in chronic diseases; indeed, clinical trials and experimental studies show that anti-IL-1 antibodies and COX-2 inhibitors improve anorexia in cancer cachexia and systemic inflammation (Bottcher et al., 2020).

While a direct role of tanycytes in thermal regulation is currently unexplored, (i) its unique position to transfer information between the liquor- and the vascular space and (ii) its connectivity to hypothalamic homeostasis centers likely distinguishes tanycytes as excellent candidates for key players in thermal/dietetic regulation. Tanycytes carry TRPV1; this non-selective cation channel can be activated by noxious high temperatures (> 42°C), pH, positive voltages, and chemicals such as capsaicin (Zhou et al., 2024). Both peripheral and central application of the TRPV1 agonist resiniferatoxin induces c-Fos expression in tanycytes (Mannari et al., 2013), which parallels STAT3 signaling activation in tanycytes but less so in neurons, suggesting an emerging role of tanycytes in thermoregulation (Mannari et al., 2013) as “warm-sensitive glial cells.” This mechanism may contribute to hypothermia, which is triggered by central administration of TRPV1 agonists (Miyata, 2022) and realized by the intimate relation between tanycytes and neurons, and by corresponding information flow: opsin 5-, adcyap1-, or QRFP-expressing warm-sensitive neurons can likely be activated by tanycytes. A further, indirect mechanism emerges via a Na^+^-dependent release of the glial transmitter lactate from tanycytes; in this case, increased Na^+^ level is due to increased ambient temperature and consequential fluid loss. Tanycytic lactate then stimulates GABAergic neurons in the subfornical organ to induce water intake behavior (Miyata, 2022). Tanycytes can also react to annual changes of the ambient temperature, which impact gonadal development and seasonal timing (but is not related, at least directly, to food intake): in common voles (*Microtus arvalis*), the expressions of types 2 and 3 of the deiodinase enzime in tanycytes associate with the summer and winter periods, respectively. Higher type 3 deiodinase enzyme levels in warm spring reduce T3 levels, ultimately suppressing gonadal development (van Rosmalen et al., 2021).

## Regenerative Capacity of Food-Intake and Thermoregulatory Mechanisms in the Hypothalamus

In general, the adult brain has a poor regenerative potential, principally dependent on its critically limited neurogenesis. Nevertheless, in addition to the subgranular zone in the hippocampal dentate gyrus and the subventricular zone of the lateral ventricles, newborn cells also emerge in the hypothalamus in adult vertebrates (Perez-Martin et al., 2010; Makrygianni and Chrousos, 2023). The first, rather ambiguous, description of a hypothalamic “germinal matrix” in adult rats was followed by later studies, which identified the source of adult-born neurons in the subependymal layer of the third ventricle neighboring the hypothalamus (Perez-Martin et al., 2010). A recent fate-mapping experiment using transgenic mouse lines expressing reporter genes under the control of tanycytic promoters showed that tanycytes, in addition to their functional and morphological plasticity, display neural stem cell properties (Prevot et al., 2018). Tanycytes can proliferate *in vivo*, and fibroblast growth factor 10-producing tanycytes can produce neurons, which populate the arcuate nucleus, although this is not observed in early postnatal tanycytes (Haan et al., 2013). Whereas the molecular control of these specific tanycyte populations remains obscure, the typical proliferation and neurogenesis regulators fibroblast growth factors 1 and 2 and IGF-1 stimulate the proliferation of arcuate nucleus neuron-giving tanycytes (Perez-Martin et al., 2010; Esteve et al., 2024) and intracerebroventricular administration of IGF-1 stimulates hypothalamic neurogenesis (Perez-Martin et al., 2010). Earlier studies suggested that non-physiological levels of growth factors increased hypothalamic neurogenesis: fibroblast growth factor and CNTF increased bromodeoxyuridine incorporation in the hypothalamus of adult rats (Perez-Martin et al., 2010).

Nevertheless, it soon emerged that adult neurogenesis also occurs under baseline conditions in the hypothalamus. The turnover of anorexigenic POMC neurons and orexigenic NPY neurons is remarkably dynamic in the arcuate nucleus of the adult mouse: during the second and third postnatal months, over half of both neuron populations are replaced (McNay et al., 2012). Remarkably, tanycytes of the hypothalamic median eminence and arcuate nucleus form a diet-responsive neurogenic niche (Lee et al., 2012). Thus, high-fat diet induced adult neurogenesis in the median eminence, and blocking this neurogenesis altered the weight and metabolic activity of adult mice (Lee et al., 2012). In the arcuate nucleus, the newly born POMC and NPY neurons are the target of a diet-induced circuit control: diet-induced obesity selectively triggers apoptosis in these newly divided cells, which can be reversed by a short period of calory restriction (McNay et al., 2012). Furthermore, diet-induced obesity leads to the depletion of actively proliferating progenitor-like cells in the hypothalamus (McNay et al., 2012). In this way, diet-induced obesity causes a relative ageing in the hypothalamus by destroying neurogenesis and neural differentiation in the arcuate nucleus. Leptin emerged as a critical neurohormone in this regulatory mechanism: in the *ob*/*ob* genetic mouse model of obesity, neurogenesis is severely reduced because of the lack of leptin, as shown by a widespread loss of the neural stem cell markers glial fibrillary acidic protein, vimentin, nestin, and Sox2 in the hypothalamus (McNay et al., 2012). Astonishingly, neurogenesis is shaped at a system-level in the hypothalamus: the high-fat-diet-induced neurogenesis in the median eminence is paralleled by the inhibition of neurogenesis in the arcuate nucleus (McNay et al., 2012).

Diet-induced obesity can impair homeostatic circuitries in the hypothalamus critical for food intake already at a very early age and even prenatally: perinatal obesogenic conditions such as maternal obesity or postnatal overnutrition induce a metabolic inflammation in the offspring hypothalamus (Park et al., 2020), characterized by the involvement of a wealth of inflammatory cytokines (Ullah et al., 2021). The process likely involves glial cells, which not only secrete but also activate inflammatory mediators in POMC and AgRP neurons (Dalvi et al., 2017). Of note, the resulting mild inflammation leads to leptin and insulin resistance, and it also reduces the expression of neurogenic factors and axon-guiding molecules. Arcuate AgRP and POMC neurons project to different nuclei of the hypothalamus, including the lateral hypothalamus and dorsomedial and paraventricular nuclei, to exert their metabolic effect. In the offspring of diabetic mothers, perinatal metabolic inflammation, similar to early leptin resistance, diminishes the projection of both AgRP and POMC neurons onto the paraventricular nucleus within the hypothalamus (Steculorum and Bouret, 2011). Furthermore, the number of AGRP and POMC neurons increases and decreases in the arcuate nucleus, respectively (Franke et al., 2005), which aggravates a disbalance in the hypothalamic feeding control circuitry. Since leptin-deficient *ob*/*ob* mice show similar neuroanatomical changes but intracerebroventricular administration of leptin restores disrupted AGRP/POMC neurons’ projections (Bouret et al., 2004), the establishment of the arcuate neuron circuitries is critically dependent on leptin signaling (Skoracka et al., 2025).

The regenerative capacity of hypothalamic centers critical for thermoregulation and food-intake/fasting also contributes to heat acclimation, an adaptive physiological process that increases heat tolerance of the individual (Ambroziak et al., 2025; Li et al., 2025b). While short-term heat exposure does not facilitate adult neurogenesis, long-term heat acclimation needs structural renewal; in addition to morphological, synaptic, and epigenetic changes, newly-born neurons are included into the hypothalamic circuitry (Armstrong and Stoppani, 2002). Neural stem- and progenitor cells derive from the ependymal layer of the third ventricle and migrate to the hypothalamic parenchyma. Here, they undergo neuronal maturation and functionally integrate into neural networks. Thus, constant moderate heat exposure for 5 days increased the number of newborn neurons in the rat hypothalamus (Matsuzaki et al., 2017) and continued heat exposure for weeks promoted their neuronal differentiation and incorporation into the local circuitry (Matsuzaki et al., 2009). This acquired heat acclimation is, however, age-dependent: long-term high ambient temperature for 40–50 days caused a greater increase in abdominal temperature with advanced age (Matsuzaki et al., 2015). Reduced heat acclimation was paralleled with a reduced number of hypothalamic bromodeoxyuridine-immunopositive cells double-stained with a mature neuron marker, neuronal nuclei (Matsuzaki et al., 2015). Thus, acquired heat acclimation likely depends on chronic heat-exposure-induced neural stem cell proliferation and integration, especially since their inhibition by a mitotic blocker, cytosine arabinoside, decreased heat tolerance in rats (Matsuzaki et al., 2017). Newborn differentiated forebrain neurons, similarly to their adult preoptic temperature-sensitive neuron counterparts, sense temperature directly: mild heat exposure promoted proliferation and neuronal differentiation of rat forebrain neural stem/progenitor cells propagated as neurospheres through activation of the Akt pathway (Hossain et al., 2017).

Heat stress itself has long been acknowledged as fatal for neurogenesis: early studies showed that thermal stress in embryonic development results in widespread organ injuries, amongst which the brain is most dramatically affected due to defunct proliferation of neuroblasts (Upfold et al., 1989). A devastating effect of higher ambient temperature has also been confirmed in the adult brain: a four-hour exposure to 38 °C triggered cell damage in several brain regions of the rat brain (Sharma et al., 2003). Heat stress, at the same time, attenuates new cell generation in the hypothalamus, at least in chicks. This affects the critical period of thermal-control establishment: High, but not mild, ambient temperatures for 24 hours diminished the number of newborn cells in the anterior hypothalamus in 3-day-old chicks one week after treatment (Kisliouk et al., 2014). Moreover, the heat challenge of previously high-temperature-conditioned chicks abolished hypothalamic neurogenesis (Kisliouk et al., 2014). The underlying mechanism likely includes miR-138, a microRNA, which promotes hypothalamic cell migration *in vitro*. Similarly, pharmaceutical blockade of thermogenesis by using the antipyretic drug paracetamol (N-acetyl-para-aminophenol) inhibits adult neurogenesis in rats: its overdose for 3–4 consecutive days reduces the number of newborn neurons in the hypothalamus two weeks after treatment (Suarez et al., 2024).

An intriguing question remains, whether in addition to the increase of ambient temperature, the rise of body core temperature could also trigger neural changes in the hypothalamus, which could subsequently improve homeostatic regulation. Nevertheless, previous investigations in other neuronal systems showed that differentiation and complexity of neurons can positively depend on thermal challenge. Thus, hyperthermia influences fate determination: it promotes neuronal and glial differentiation of neural stem cells as shown by flow cytometry and Western Blotting of the neuronal marker Tuj-1, the glial cell marker glial fibrillary acidic protein as well as LncRNAs – expressed during the neural development – of cultured rat neural stem cells (Wang et al., 2017). An extreme form of fever-associated pathological state, febrile seizure, increases dendritic complexity of newborn dentate granule cells in early postnatal life, at least in rats (Raijmakers et al., 2016). Even prolonged febrile seizures did not affect neurogenesis (nor neuronal loss) but increased mossy fiber plasticity and enhanced hippocampal excitability due to axonal reorganization and proliferation (Bender et al., 2003). In the hypothalamus, a previous study suggested that fever-like induced neurogenesis could affect body weight control (Solymar et al., 2011). CNTF, an inducer of neurogenesis in the hypothalamus, causes a permanent loss of body mass in mice made obese by a fat-rich diet (Schuster et al., 2003). A 7-day-long intracerebroventricular infusion of CNTF, simulating a fever-like state, in freely moving mice with diet-induced obesity reduced body mass beyond the period of infusion, which was paralleled by increased abdominal core temperature and reduced locomotor activity (Solymar et al., 2011). While direct evidence is lacking, these results support the idea that CNTF-induced neurogenesis in the hypothalamus could decrease body mass in obese subjects.

## Conclusions

The dedicated neuronal ensembles, which regulate food intake or body temperature do not act as separate players of isolated homeostatic systems. Instead, the well-defined nuclei and cell types of select brain centers responsible for food intake, fasting, and warm- or cold sensation receive a wealth of signals informing them about the cross-modalities of homeostasis. Further, in addition to processing thermal or dietetic information and triggering adequate actions within their own systems, they can also contribute to the other systems by supplying useful information and shaping functions. In addition to the tandem mechanisms between thermoregulation and food-intake regulation, various modulatory inputs also intervene in the system. These include blood- and liquor-borne signals, and non-neuronal elements such as tanycytes and glia may be used to convey information. The druggable targets of this manifold puzzle offer intervention points in this delicately balanced, vulnerable network.

**Additional file:** *Open peer review report 1.*

OPEN PEER REVIEW REPORT 1

## Data Availability

*All relevant data are within the paper and its Additional files.*

## References

[R1] Abd El Hay MY, Kamm GB, Tlaie Boria A, Siemens J (2025). Diverging roles of TRPV1 and TRPM2 in warm-temperature detection. Elife.

[R2] Ambroziak W, Nencini S, Pohle J, Zuza K, Pino G, Lundh S, Araujo-Sousa C, Goetz LIL, Schrenk-Siemens K, Manoj G, Herrera MA, Acuna C, Siemens J (2025). Thermally induced neuronal plasticity in the hypothalamus mediates heat tolerance. Nat Neurosci.

[R3] Armstrong LE, Stoppani J (2002). Central nervous system control of heat acclimation adaptations: an emerging paradigm. Rev Neurosci.

[R4] Barrios V, Frago LM, Canelles S, Guerra-Cantera S, Arilla-Ferreiro E, Chowen JA, Argente J (2021). Leptin modulates the response of brown adipose tissue to negative energy balance: implication of the GH/IGF-I axis. Int J Mol Sci.

[R5] Bein T (2024). Pathophysiology and management of heat illness. Med Klin Intensivmed Notfmed.

[R6] Bender RA, Dube C, Gonzalez-Vega R, Mina EW, Baram TZ (2003). Mossy fiber plasticity and enhanced hippocampal excitability, without hippocampal cell loss or altered neurogenesis, in an animal model of prolonged febrile seizures. Hippocampus.

[R7] Benevento M, Alpar A, Gundacker A, Afjehi L, Balueva K, Hevesi Z, Hanics J, Rehman S, Pollak DD, Lubec G, Wulff P, Prevot V, Horvath TL, Harkany T (2024). A brainstem-hypothalamus neuronal circuit reduces feeding upon heat exposure. Nature.

[R8] Benford H, Bolborea M, Pollatzek E, Lossow K, Hermans-Borgmeyer I, Liu B, Meyerhof W, Kasparov S, Dale N (2017). A sweet taste receptor-dependent mechanism of glucosensing in hypothalamic tanycytes. Glia.

[R9] Bernhard MC, Li P, Allison DB, Gohlke JM (2015). Warm ambient temperature decreases food intake in a simulated office setting: a pilot randomized controlled trial. Front Nutr.

[R10] Berthoud HR (2002). Multiple neural systems controlling food intake and body weight. Neurosci Biobehav Rev.

[R11] Bombassaro B, Araujo EP, Velloso LA (2024). The hypothalamus as the central regulator of energy balance and its impact on current and future obesity treatments. Arch Endocrinol Metab.

[R12] Bottcher M, Muller-Fielitz H, Sundaram SM, Gallet S, Neve V, Shionoya K, Zager A, Quan N, Liu X, Schmidt-Ullrich R, Haenold R, Wenzel J, Blomqvist A, Engblom D, Prevot V, Schwaninger M (2020). NF-kappaB signaling in tanycytes mediates inflammation-induced anorexia. Mol Metab.

[R13] Bouret SG, Draper SJ, Simerly RB (2004). Trophic action of leptin on hypothalamic neurons that regulate feeding. Science.

[R14] Brunner M, Lopez-Rodriguez D, Estrada-Meza J, Dali R, Rohrbach A, Deglise T, Messina A, Thorens B, Santoni F, Langlet F (2024). Fasting induces metabolic switches and spatial redistributions of lipid processing and neuronal interactions in tanycytes. Nat Commun.

[R15] Cabral A, Valdivia S, Fernandez G, Reynaldo M, Perello M (2014). Divergent neuronal circuitries underlying acute orexigenic effects of peripheral or central ghrelin: critical role of brain accessibility. J Neuroendocrinol.

[R16] Cerri M, Morrison SF (2006). Corticotropin releasing factor increases in brown adipose tissue thermogenesis and heart rate through dorsomedial hypothalamus and medullary raphe pallidus. Neuroscience.

[R17] Chitravanshi VC, Kawabe K, Sapru HN (2016). Stimulation of the hypothalamic arcuate nucleus increases brown adipose tissue nerve activity via hypothalamic paraventricular and dorsomedial nuclei. Am J Physiol Heart Circ Physiol.

[R18] Coppola A, Liu ZW, Andrews ZB, Paradis E, Roy MC, Friedman JM, Ricquier D, Richard D, Horvath TL, Gao XB, Diano S (2007). A central thermogenic-like mechanism in feeding regulation: an interplay between arcuate nucleus T3 and UCP2. Cell Metab.

[R19] Dalvi PS, Chalmers JA, Luo V, Han DY, Wellhauser L, Liu Y, Tran DQ, Castel J, Luquet S, Wheeler MB, Belsham DD (2017). High fat induces acute and chronic inflammation in the hypothalamus: effect of high-fat diet, palmitate and TNF-alpha on appetite-regulating NPY neurons. Int J Obes.

[R20] Dangarembizi R, Erlwanger KH, Rummel C, Roth J, Madziva MT, Harden LM (2018). Brewer’s yeast is a potent inducer of fever, sickness behavior and inflammation within the brain. Brain Behav Immun.

[R21] De Jonghe BC, Hayes MR, Banno R, Skibicka KP, Zimmer DJ, Bowen KA, Leichner TM, Alhadeff AL, Kanoski SE, Cyr NE, Nillni EA, Grill HJ, Bence KK (2011). Deficiency of PTP1B in POMC neurons leads to alterations in energy balance and homeostatic response to cold exposure. Am J Physiol Endocrinol Metab.

[R22] Deem JD, Faber CL, Pedersen C, Phan BA, Larsen SA, Ogimoto K, Nelson JT, Damian V, Tran MA, Palmiter RD, Kaiyala KJ, Scarlett JM, Bruchas MR, Schwartz MW, Morton GJ (2020). Cold-induced hyperphagia requires AgRP neuron activation in mice. Elife.

[R23] Deem JD, Tingley D, Watts CA, Ogimoto K, Bryan CL, Phan BAN, Damian V, Bruchas MR, Scarlett JM, Schwartz MW, Morton GJ (2023). High-fat diet feeding disrupts the coupling of thermoregulation to energy homeostasis. Mol Metab.

[R24] Delezie J, Gill JF, Santos G, Karrer-Cardel B, Handschin C (2020). PGC-1beta-expressing POMC neurons mediate the effect of leptin on thermoregulation in the mouse. Sci Rep.

[R25] Deng J, Yuan F, Guo Y, Xiao Y, Niu Y, Deng Y, Han X, Guan Y, Chen S, Guo F (2017). Deletion of ATF4 in AgRP neurons promotes fat loss mainly via increasing energy expenditure. Diabetes.

[R26] Dodd GT, Andrews ZB, Simonds SE, Michael NJ, DeVeer M, Bruning JC, Spanswick D, Cowley MA, Tiganis T (2017). A hypothalamic phosphatase switch coordinates energy expenditure with feeding. Cell Metab.

[R27] Dong Y, Carty J, Goldstein N, He Z, Hwang E, Chau D, Wallace B, Kabahizi A, Lieu L, Peng Y, Gao Y, Hu L, Betley JN, Williams KW (2021). Time and metabolic state-dependent effects of GLP-1R agonists on NPY/AgRP and POMC neuronal activity in vivo. Mol Metab.

[R28] Dubins JS, Sanchez-Alavez M, Zhukov V, Sanchez-Gonzalez A, Moroncini G, Carvajal-Gonzalez S, Hadcock JR, Bartfai T, Conti B (2012). Downregulation of GPR83 in the hypothalamic preoptic area reduces core body temperature and elevates circulating levels of adiponectin. Metabolism.

[R29] Duquenne M, Deligia E, Folgueira C, Bourouh C, Caron E, Pfrieger F, Schwaninger M, Nogueiras R, Annicotte JS, Imbernon M, Prevot V (2024). Tanycytic transcytosis inhibition disrupts energy balance, glucose homeostasis and cognitive function in male mice. Mol Metab.

[R30] Esteve NA, Rogers DJ, Stagray JA, Mayeux H, Nora G, Huval L, Smith KM (2024). Tanycyte radial morphology and proliferation are influenced by fibroblast growth factor receptor 1 and high-fat diet. Eur J Neurosci.

[R31] Faouzi M, Leshan R, Bjornholm M, Hennessey T, Jones J, Munzberg H (2007). Differential accessibility of circulating leptin to individual hypothalamic sites. Endocrinology.

[R32] Feketa VV, Nikolaev YA, Merriman DK, Bagriantsev SN, Gracheva EO (2020). CNGA3 acts as a cold sensor in hypothalamic neurons. Elife.

[R33] Fioramonti X, Contie S, Song Z, Routh VH, Lorsignol A, Penicaud L (2007). Characterization of glucosensing neuron subpopulations in the arcuate nucleus: integration in neuropeptide Y and pro-opio melanocortin networks?. Diabetes.

[R34] Florant GL, Nuttle LC, Mullinex DE, Rintoul DA (1990). Plasma and white adipose tissue lipid composition in marmots. Am J Physiol.

[R35] Florant GL, Healy JE (2012). The regulation of food intake in mammalian hibernators: a review. J Comp Physiol B.

[R36] Francois M, Kaiser L, He Y, Xu Y, Salbaum JM, Yu S, Morrison CD, Berthoud HR, Munzberg H (2025). Leptin receptor neurons in the dorsomedial hypothalamus require distinct neuronal subsets for thermogenesis and weight loss. Metabolism.

[R37] Franke K, Harder T, Aerts L, Melchior K, Fahrenkrog S, Rodekamp E, Ziska T, Van Assche FA, Dudenhausen JW, Plagemann A (2005). ‘Programming’ of orexigenic and anorexigenic hypothalamic neurons in offspring of treated and untreated diabetic mother rats. Brain Res.

[R38] Frare C, Drew KL (2021). Seasonal changes in adenosine kinase in tanycytes of the Arctic ground squirrel (Urocitellus parryii). J Chem Neuroanat.

[R39] Frayling C, Britton R, Dale N (2011). ATP-mediated glucosensing by hypothalamic tanycytes. J Physiol.

[R40] Frontini A, Giordano A (2010). Leptin-sensitive neurons in mouse preoptic area express alpha 1A- and alpha 2A-adrenergic receptor isoforms. Neurosci Lett.

[R41] Fuller A, Carter RN, Mitchell D (1998). Brain and abdominal temperatures at fatigue in rats exercising in the heat. J Appl Physiol.

[R42] Gomez IM, Uriarte M, Fernandez G, Barrile F, Castrogiovanni D, Cantel S, Fehrentz JA, De Francesco PN, Perello M (2024). Hypothalamic tanycytes internalize ghrelin from the cerebrospinal fluid: molecular mechanisms and functional implications. Mol Metab.

[R43] Groh AMR, Song YL, Tea F, Lu B, Huynh S, Afanasiev E, Bigotte M, Del Bigio MR, Stratton JA (2024). Multiciliated ependymal cells: an update on biology and pathology in the adult brain. Acta Neuropathol.

[R44] Haan N, Goodman T, Najdi-Samiei A, Stratford CM, Rice R, El Agha E, Bellusci S, Hajihosseini MK (2013). Fgf10-expressing tanycytes add new neurons to the appetite/energy-balance regulating centers of the postnatal and adult hypothalamus. J Neurosci.

[R45] Halawi H, Khemani D, Eckert D, O’Neill J, Kadouh H, Grothe K, Clark MM, Burton DD, Vella A, Acosta A, Zinsmeister AR, Camilleri M (2017). Effects of liraglutide on weight, satiation, and gastric functions in obesity: a randomised, placebo-controlled pilot trial. Lancet Gastroenterol Hepatol.

[R46] Hossain ME, Matsuzaki K, Katakura M, Sugimoto N, Mamun AA, Islam R, Hashimoto M, Shido O (2017). Direct exposure to mild heat promotes proliferation and neuronal differentiation of neural stem/progenitor cells in vitro. PLoS One.

[R47] Huffer K, Denley MCS, Oskoui EV, Swartz KJ (2024). Conservation of the cooling agent binding pocket within the TRPM subfamily. Elife.

[R48] Imbernon M, Saponaro C, Helms HCC, Duquenne M, Fernandois D, Deligia E, Denis RGP, Chao DHM, Rasika S, Staels B, Pattou F, Pfrieger FW, Brodin B, Luquet S, Bonner C, Prevot V (2022). Tanycytes control hypothalamic liraglutide uptake and its anti-obesity actions. Cell Metab.

[R49] Jeong JH, Lee DK, Liu SM, Chua SC, Schwartz GJ, Jo YH (2018). Activation of temperature-sensitive TRPV1-like receptors in ARC POMC neurons reduces food intake. PLoS Biol.

[R50] Kataoka N, Hioki H, Kaneko T, Nakamura K (2014). Psychological stress activates a dorsomedial hypothalamus-medullary raphe circuit driving brown adipose tissue thermogenesis and hyperthermia. Cell Metab.

[R51] Kemen J, Schaffer-Gemein S, Grunewald J, Kistemann T (2021). Heat perception and coping strategies: a structured interview-based study of elderly people in Cologne, Germany. Int J Environ Res Public Health.

[R52] Kisliouk T, Cramer T, Meiri N (2014). Heat stress attenuates new cell generation in the hypothalamus: a role for miR-138. Neuroscience.

[R53] Klein I, Sanchez-Alavez M, Tabarean I, Schaefer J, Holmberg KH, Klaus J, Xia F, Marcondes MC, Dubins JS, Morrison B, Zhukov V, Sanchez-Gonzalez A, Mitsukawa K, Hadcock JR, Bartfai T, Conti B (2011). AdipoR1 and 2 are expressed on warm sensitive neurons of the hypothalamic preoptic area and contribute to central hyperthermic effects of adiponectin. Brain Res.

[R54] Kroeger D, Absi G, Gagliardi C, Bandaru SS, Madara JC, Ferrari LL, Arrigoni E, Munzberg H, Scammell TE, Saper CB, Vetrivelan R (2018). Galanin neurons in the ventrolateral preoptic area promote sleep and heat loss in mice. Nat Commun.

[R55] Kuperman Y, Weiss M, Dine J, Staikin K, Golani O, Ramot A, Nahum T, Kuhne C, Shemesh Y, Wurst W, Harmelin A, Deussing JM, Eder M, Chen A (2016). CRFR1 in AgRP neurons modulates sympathetic nervous system activity to adapt to cold stress and fasting. Cell Metab.

[R56] Langlet F, Levin BE, Luquet S, Mazzone M, Messina A, Dunn-Meynell AA, Balland E, Lacombe A, Mazur D, Carmeliet P, Bouret SG, Prevot V, Dehouck B (2013). Tanycytic VEGF-A boosts blood-hypothalamus barrier plasticity and access of metabolic signals to the arcuate nucleus in response to fasting. Cell Metab.

[R57] Lee DA, Bedont JL, Pak T, Wang H, Song J, Miranda-Angulo A, Takiar V, Charubhumi V, Balordi F, Takebayashi H, Aja S, Ford E, Fishell G, Blackshaw S (2012). Tanycytes of the hypothalamic median eminence form a diet-responsive neurogenic niche. Nat Neurosci.

[R58] Lee J, Raycraft L, Johnson AW (2021). The dynamic regulation of appetitive behavior through lateral hypothalamic orexin and melanin concentrating hormone expressing cells. Physiol Behav.

[R59] Leva TM, Whitmire CJ (2023). Thermosensory thalamus: parallel processing across model organisms. Front Neurosci.

[R60] Lewis S (2025). Shaping preoptic-area neuronal diversity. Nat Rev Neurosci.

[R61] Li D, Zou B, Hu Q, Zhang X, Zeng W, Liu L, Yu M (2025). Single-cell RNA sequencing of the primary visual cortex in mice with optic nerve injury. Invest Ophthalmol Vis Sci.

[R62] Li J, Zhou Z, Wu Y, Zhao J, Duan H, Peng Y, Wang X, Fan Z, Yin L, Li M, Liu F, Yang Y, Du L, Li J, Zhong H, Hou W, Zhang F, Ma H, Zhang X (2025). Heat acclimation defense against exertional heat stroke by improving the function of preoptic TRPV1 neurons. Theranostics.

[R63] Liu J, Varghese BM, Hansen A, Zhang Y, Driscoll T, Morgan G, Dear K, Gourley M, Capon A, Bi P (2022). Heat exposure and cardiovascular health outcomes: a systematic review and meta-analysis. Lancet Planet Health.

[R64] Machado NLS, Saper CB (2022). Genetic identification of preoptic neurons that regulate body temperature in mice. Temperature (Austin).

[R65] Makrygianni EA, Chrousos GP (2023). Neural progenitor cells and the hypothalamus. Cells.

[R66] Mannari T, Morita S, Furube E, Tominaga M, Miyata S (2013). Astrocytic TRPV1 ion channels detect blood-borne signals in the sensory circumventricular organs of adult mouse brains. Glia.

[R67] Matsuzaki K, Katakura M, Hara T, Li G, Hashimoto M, Shido O (2009). Proliferation of neuronal progenitor cells and neuronal differentiation in the hypothalamus are enhanced in heat-acclimated rats. Pflugers Arch.

[R68] Matsuzaki K, Katakura M, Inoue T, Hara T, Hashimoto M, Shido O (2015). Aging attenuates acquired heat tolerance and hypothalamic neurogenesis in rats. J Comp Neurol.

[R69] Matsuzaki K, Katakura M, Sugimoto N, Hara T, Hashimoto M, Shido O (2017). Neural progenitor cell proliferation in the hypothalamus is involved in acquired heat tolerance in long-term heat-acclimated rats. PLoS One.

[R70] McAllen RM, McKinley MJ (2018). Efferent thermoregulatory pathways regulating cutaneous blood flow and sweating. Handb Clin Neurol.

[R71] McKinley MJ, Pennington GL, Ryan PJ (2021). The median preoptic nucleus: a major regulator of fluid, temperature, sleep, and cardiovascular homeostasis. Handb Clin Neurol.

[R72] McNay DE, Briancon N, Kokoeva MV, Maratos-Flier E, Flier JS (2012). Remodeling of the arcuate nucleus energy-balance circuit is inhibited in obese mice. J Clin Invest.

[R73] Miyata S (2022). Glial functions in the blood-brain communication at the circumventricular organs. Front Neurosci.

[R74] Mondino A, Jadidian A, Toth BA, Hambrecht-Wiedbusch VS, Floran-Garduno L, Li D, York AK, Torterolo P, Pal D, Burgess CR, Mashour GA, Vanini G (2025). Regulation of REM and NREM sleep by preoptic glutamatergic neurons. Sleep.

[R75] Morrison SF, Nakamura K (2019). Central mechanisms for thermoregulation. Annu Rev Physiol.

[R76] Morrison SF (2004). Activation of 5-HT1A receptors in raphe pallidus inhibits leptin-evoked increases in brown adipose tissue thermogenesis. Am J Physiol Regul Integr Comp Physiol.

[R77] Mota CMD, Madden CJ (2024). Neural circuits of long-term thermoregulatory adaptations to cold temperatures and metabolic demands. Nat Rev Neurosci.

[R78] Nakamura K, Morrison SF (2010). A thermosensory pathway mediating heat-defense responses. Proc Natl Acad Sci U S A.

[R79] Nakamura K, Nakamura Y, Kataoka N (2022). A hypothalamomedullary network for physiological responses to environmental stresses. Nat Rev Neurosci.

[R80] Nakamura K (2024). Central mechanisms of thermoregulation and fever in mammals. Adv Exp Med Biol.

[R81] Nakamura Y, Nakamura K, Matsumura K, Kobayashi S, Kaneko T, Morrison SF (2005). Direct pyrogenic input from prostaglandin EP3 receptor-expressing preoptic neurons to the dorsomedial hypothalamus. Eur J Neurosci.

[R82] Nakamura Y, Yahiro T, Fukushima A, Kataoka N, Hioki H, Nakamura K (2022). Prostaglandin EP3 receptor-expressing preoptic neurons bidirectionally control body temperature via tonic GABAergic signaling. Sci Adv.

[R83] Norris AJ, Shaker JR, Cone AL, Ndiokho IB, Bruchas MR (2021). Parabrachial opioidergic projections to preoptic hypothalamus mediate behavioral and physiological thermal defenses. Elife.

[R84] Notley SR, Mitchell D, Taylor NAS (2023). A century of exercise physiology: concepts that ignited the study of human thermoregulation. Part 1: Foundational principles and theories of regulation. Eur J Appl Physiol.

[R85] Osaka T (2011). Hypoxia-induced hypothermia mediated by noradrenaline and nitric oxide in the rostromedial preoptic area. Neuroscience.

[R86] Osaka T (2022). The EP(3) and EP(4) receptor subtypes both mediate the fever-producing effects of prostaglandin E(2) in the rostral ventromedial preoptic area of the hypothalamus in rats. Neuroscience.

[R87] Osborn O, Sanchez-Alavez M, Dubins JS, Gonzalez AS, Morrison B, Hadcock JR, Bartfai T (2011). Ccl22/MDC, is a prostaglandin dependent pyrogen, acting in the anterior hypothalamus to induce hyperthermia via activation of brown adipose tissue. Cytokine.

[R88] Palmiter RD (2018). The parabrachial nucleus: CGRP neurons function as a general alarm. Trends Neurosci.

[R89] Park S, Jang A, Bouret SG (2020). Maternal obesity-induced endoplasmic reticulum stress causes metabolic alterations and abnormal hypothalamic development in the offspring. PLoS Biol.

[R90] Pauli JL, Chen JY, Basiri ML, Park S, Carter ME, Sanz E, McKnight GS, Stuber GD, Palmiter RD (2022). Molecular and anatomical characterization of parabrachial neurons and their axonal projections. Elife.

[R91] Peier AM, Moqrich A, Hergarden AC, Reeve AJ, Andersson DA, Story GM, Earley TJ, Dragoni I, McIntyre P, Bevan S, Patapoutian A (2002). A TRP channel that senses cold stimuli and menthol. Cell.

[R92] Perez-Martin M, Cifuentes M, Grondona JM, Lopez-Avalos MD, Gomez-Pinedo U, Garcia-Verdugo JM, Fernandez-Llebrez P (2010). IGF-I stimulates neurogenesis in the hypothalamus of adult rats. Eur J Neurosci.

[R93] Pfau SJ, Langen UH, Fisher TM, Prakash I, Nagpurwala F, Lozoya RA, Lee WA, Wu Z, Gu C (2024). Characteristics of blood-brain barrier heterogeneity between brain regions revealed by profiling vascular and perivascular cells. Nat Neurosci.

[R94] Pinol RA, Mogul AS, Hadley CK, Saha A, Li C, Skop V, Province HS, Xiao C, Gavrilova O, Krashes MJ, Reitman ML (2021). Preoptic BRS3 neurons increase body temperature and heart rate via multiple pathways. Cell Metab.

[R95] Prevot V, Dehouck B, Sharif A, Ciofi P, Giacobini P, Clasadonte J (2018). The versatile tanycyte: a hypothalamic integrator of reproduction and energy metabolism. Endocr Rev.

[R96] Prevot V, Nogueiras R, Schwaninger M (2021). Tanycytes in the infundibular nucleus and median eminence and their role in the blood-brain barrier. Handb Clin Neurol.

[R97] Qian S, Yan S, Pang R, Zhang J, Liu K, Shi Z, Wang Z, Chen P, Zhang Y, Luo T, Hu X, Xiong Y, Zhou Y (2022). A temperature-regulated circuit for feeding behavior. Nat Commun.

[R98] Quinones M (2018). p53 in AgRP neurons is required for protection against diet-induced obesity via JNK1. Nat Commun.

[R99] Raijmakers M, Clynen E, Smisdom N, Nelissen S, Brone B, Rigo JM, Hoogland G, Swijsen A (2016). Experimental febrile seizures increase dendritic complexity of newborn dentate granule cells. Epilepsia.

[R100] Reyes TM, Sawchenko PE (2002). Involvement of the arcuate nucleus of the hypothalamus in interleukin-1-induced anorexia. J Neurosci.

[R101] Ribeiro FM, Arnaldo L, P Milhomem L, S Aguiar S, Franco OL (2025). The intricate relationship between circadian rhythms and gastrointestinal peptides in obesity. Peptides.

[R102] Rogers JF, Vandendoren M, Prather JF, Landen JG, Bedford NL, Nelson AC (2024). Neural cell-types and circuits linking thermoregulation and social behavior. Neurosci Biobehav Rev.

[R103] Rothhaas R, Chung S (2021). Role of the preoptic area in sleep and thermoregulation. Front Neurosci.

[R104] Ruan HB, Dietrich MO, Liu ZW, Zimmer MR, Li MD, Singh JP, Zhang K, Yin R, Wu J, Horvath TL, Yang X (2014). O-GlcNAc transferase enables AgRP neurons to suppress browning of white fat. Cell.

[R105] Saito M, Matsushita M, Yoneshiro T, Okamatsu-Ogura Y (2020). Brown adipose tissue, diet-induced thermogenesis, and thermogenic food ingredients: from mice to men. Front Endocrinol (Lausanne).

[R106] Salgado M, Garcia-Robles MA, Saez JC (2021). Purinergic signaling in tanycytes and its contribution to nutritional sensing. Purinergic Signal.

[R107] Sanchez-Alavez M, Osborn O, Tabarean IV, Holmberg KH, Eberwine J, Kahn CR, Bartfai T (2011). Insulin-like growth factor 1-mediated hyperthermia involves anterior hypothalamic insulin receptors. J Biol Chem.

[R108] Schuster B, Kovaleva M, Sun Y, Regenhard P, Matthews V, Grotzinger J, Rose-John S, Kallen KJ (2003). Signaling of human ciliary neurotrophic factor (CNTF) revisited. The interleukin-6 receptor can serve as an alpha-receptor for CTNF. J Biol Chem.

[R109] Secher A, Jelsing J, Baquero AF, Hecksher-Sorensen J, Cowley MA, Dalboge LS, Hansen G, Grove KL, Pyke C, Raun K, Schaffer L, Tang-Christensen M, Verma S, Witgen BM, Vrang N, Bjerre Knudsen L (2014). The arcuate nucleus mediates GLP-1 receptor agonist liraglutide-dependent weight loss. J Clin Invest.

[R110] Sharma HS, Drieu K, Westman J (2003). Antioxidant compounds EGB-761 and BN-52021 attenuate brain edema formation and hemeoxygenase expression following hyperthermic brain injury in the rat. Acta Neurochir Suppl.

[R111] Shen M, Shang M, Tian R, Hu Y, Han Q, Hu J, An G, Wang B, Cao Z, Lin X, Yang H, Xing J (2023). Single cell molecular alterations reveal target cells and pathways of conditioned fear memory. Brain Res.

[R112] Shi YC, Lau J, Lin Z, Zhang H, Zhai L, Sperk G, Heilbronn R, Mietzsch M, Weger S, Huang XF, Enriquez RF, Baldock PA, Zhang L, Sainsbury A, Herzog H, Lin S (2013). Arcuate NPY controls sympathetic output and BAT function via a relay of tyrosine hydroxylase neurons in the PVN. Cell Metab.

[R113] Sisodiya SM (2024). Hot brain: practical climate change advice for neurologists. Pract Neurol.

[R114] Skibicka KP, Alhadeff AL, Leichner TM, Grill HJ (2011). Neural controls of prostaglandin 2 pyrogenic, tachycardic, and anorexic actions are anatomically distributed. Endocrinology.

[R115] Skoracka K, Hryhorowicz S, Schulz P, Zawada A, Ratajczak-Pawlowska AE, Rychter AM, Slomski R, Dobrowolska A, Krela-Kazmierczak I (2025). The role of leptin and ghrelin in the regulation of appetite in obesity. Peptides.

[R116] Solymar M, Szelenyi Z, Petervari E (2011). A fever-like effect of central infusion of CNTF in freely moving mice with diet-induced obesity. J Mol Neurosci.

[R117] Steculorum SM, Bouret SG (2011). Maternal diabetes compromises the organization of hypothalamic feeding circuits and impairs leptin sensitivity in offspring. Endocrinology.

[R118] Straat ME, Schinkelshoek MS, Fronczek R, Lammers GJ, Rensen PCN, Boon MR (2020). Role of brown adipose tissue in adiposity associated with narcolepsy type 1. Front Endocrinol (Lausanne).

[R119] Suarez J, de Ceglia M, Rodriguez-Pozo M, Vargas A, Santos I, Melgar-Locatelli S, Castro-Zavala A, Castilla-Ortega E, Rodriguez de Fonseca F, Decara J, Rivera P (2024). Inhibition of adult neurogenesis in male mice after repeated exposure to paracetamol overdose. Int J Mol Sci.

[R120] Sunagawa GA, Takahashi M (2016). Hypometabolism during daily torpor in mice is dominated by reduction in the sensitivity of the thermoregulatory system. Sci Rep.

[R121] Takahashi K, Yamada T, Katagiri H (2024). Inter-organ communication involved in brown adipose tissue thermogenesis. Adv Exp Med Biol.

[R122] Takahashi TM, Sunagawa GA, Soya S, Abe M, Sakurai K, Ishikawa K, Yanagisawa M, Hama H, Hasegawa E, Miyawaki A, Sakimura K, Takahashi M, Sakurai T (2020). A discrete neuronal circuit induces a hibernation-like state in rodents. Nature.

[R123] Tan CL, Cooke EK, Leib DE, Lin YC, Daly GE, Zimmerman CA, Knight ZA (2016). Warm-sensitive neurons that control body temperature. Cell.

[R124] Tan CL, Knight ZA (2018). Regulation of body temperature by the nervous system. Neuron.

[R125] Tang Q, Liu Q, Li J, Yan J, Jing X, Zhang J, Xia Y, Xu Y, Li Y, He J (2022). MANF in POMC neurons promotes brown adipose tissue thermogenesis and protects against diet-induced obesity. Diabetes.

[R126] Townsend KL, Madden CJ, Blaszkiewicz M, McDougall L, Tupone D, Lynes MD, Mishina Y, Yu P, Morrison SF, Tseng YH (2017). Reestablishment of energy balance in a male mouse model with POMC neuron deletion of BMPR1A. Endocrinology.

[R127] Tran LT, Park S, Kim SK, Lee JS, Kim KW, Kwon O (2022). Hypothalamic control of energy expenditure and thermogenesis. Exp Mol Med.

[R128] Tupone D, Madden CJ, Cano G, Morrison SF (2011). An orexinergic projection from perifornical hypothalamus to raphe pallidus increases rat brown adipose tissue thermogenesis. J Neurosci.

[R129] Uchino E, Kusumoto-Yoshida I, Kashiwadani H, Kanmura Y, Matsunaga A, Kuwaki T (2024). Identification of hypothermia-inducing neurons in the preoptic area and activation of them by isoflurane anesthesia and central injection of adenosine. J Physiol Sci.

[R130] Ullah R, Rauf N, Nabi G, Yi S, Yu-Dong Z, Fu J (2021). Mechanistic insight into high-fat diet-induced metabolic inflammation in the arcuate nucleus of the hypothalamus. Biomed Pharmacother.

[R131] Upfold JB, Smith MS, Edwards MJ (1989). Quantitative study of the effects of maternal hyperthermia on cell death and proliferation in the guinea pig brain on day 21 of pregnancy. Teratology.

[R132] van Rosmalen L, van Dalum J, Appenroth D, Roodenrijs RTM, de Wit L, Hazlerigg DG, Hut RA (2021). Mechanisms of temperature modulation in mammalian seasonal timing. FASEB J.

[R133] Wang J, Wu S, Zhan H, Bi W, Xu Y, Liang Y, Ge Y, Peng L, Jin X, Lu K, Zhao J, Gao L, He Z (2022). p38alpha in the preoptic area inhibits brown adipose tissue thermogenesis. Obesity (Silver Spring).

[R134] Wang L, Deng Y, Duan D, Sun S, Ge L, Zhuo Y, Yuan T, Wu P, Wang H, Lu M, Xia Y (2017). Hyperthermia influences fate determination of neural stem cells with lncRNAs alterations in the early differentiation. PLoS One.

[R135] Wang P, Loh KH, Wu M, Morgan DA, Schneeberger M, Yu X, Chi J, Kosse C, Kim D, Rahmouni K, Cohen P, Friedman J (2020). A leptin-BDNF pathway regulating sympathetic innervation of adipose tissue. Nature.

[R136] Wang TA, Teo CF, Akerblom M, Chen C, Tynan-La Fontaine M, Greiner VJ, Diaz A, McManus MT, Jan YN, Jan LY (2019). Thermoregulation via temperature-dependent PGD(2) production in mouse preoptic area. Neuron.

[R137] Wang TA, Chen C, Huang F, Feng S, Tien J, Braz JM, Basbaum AI, Jan YN, Jan LY (2021). TMEM16C is involved in thermoregulation and protects rodent pups from febrile seizures. Proc Natl Acad Sci U S A.

[R138] Westerman AT, Roma PG, Price RC, Dominguez JM (2010). Assessing the role of the medial preoptic area in ethanol-induced hypothermia. Neurosci Lett.

[R139] Xing X, Liu S, Liu XY, Yang M, Wang DH (2020). Cold exposure increased hypothalamic orexigenic neuropeptides but not food intake in fattening Daurian ground squirrels. Zoology (Jena).

[R140] Yadav R, Jaryal AK, Mallick HN (2017). Participation of preoptic area TRPV4 ion channel in regulation of body temperature. J Therm Biol.

[R141] Yang S, Tan YL, Wu X, Wang J, Sun J, Liu A, Gan L, Shen B, Zhang X, Fu Y, Huang J (2021). An mPOA-ARC(AgRP) pathway modulates cold-evoked eating behavior. Cell Rep.

[R142] Yarmolinsky DA, Peng Y, Pogorzala LA, Rutlin M, Hoon MA, Zuker CS (2016). Coding and plasticity in the mammalian thermosensory system. Neuron.

[R143] Yu S, Qualls-Creekmore E, Rezai-Zadeh K, Jiang Y, Berthoud HR, Morrison CD, Derbenev AV, Zsombok A, Munzberg H (2016). Glutamatergic preoptic area neurons that express leptin receptors drive temperature-dependent body weight homeostasis. J Neurosci.

[R144] Yu S, Cheng H, Francois M, Qualls-Creekmore E, Huesing C, He Y, Jiang Y, Gao H, Xu Y, Zsombok A, Derbenev AV, Nillni EA, Burk DH, Morrison CD, Berthoud HR, Munzberg H (2018). Preoptic leptin signaling modulates energy balance independent of body temperature regulation. Elife.

[R145] Zhang KX (2020). Violet-light suppression of thermogenesis by opsin 5 hypothalamic neurons. Nature.

[R146] Zhang W, Sunanaga J, Takahashi Y, Mori T, Sakurai T, Kanmura Y, Kuwaki T (2010). Orexin neurons are indispensable for stress-induced thermogenesis in mice. J Physiol.

[R147] Zhang Z, Shan L, Wang Y, Li W, Jiang M, Liang F, Feng S, Lu Z, Wang H, Dai J (2023). Primate preoptic neurons drive hypothermia and cold defense. Innovation (Camb).

[R148] Zhou Q (2023). Hypothalamic warm-sensitive neurons require TRPC4 channel for detecting internal warmth and regulating body temperature in mice. Neuron.

[R149] Zhou S, Makashova O, Chevillard PM, Josey V, Li B, Prager-Khoutorsky M (2024). Constitutive cell proliferation and neurogenesis in the organum vasculosum lamina terminalis and subfornical organ of adult rats. J Neuroendocrinol.

