# Supplementary material for: Thermal challenge and food intake: Mutual regulatory mechanisms
Source: Neural Regen Res. 2025 Sep 29;21(9):4169–76. doi: 10.4103/NRR.NRR-D-25-00170 (PMC13557685; doi:10.4103/NRR.NRR-D-25-00170)
Supplement: Supplementary file 1 [file NRR-21-4169_Suppl1.pdf]

OPEN PEER REVIEW REPORT 1

Name of journal: Neural Regeneration Research
Manuscript NO: NRR-D-25-00170
Title: The brain control of thermal challenge and food-intake: crossroads of regulatory mechanisms
Reviewer's Name: Luciane H. Gargaglioni
Reviewer's country: Brazil

COMMENTS TO AUTHORS

This review provides a comprehensive overview of the neural mechanisms underlying the regulation of thermal challenges and food intake, with a particular focus on the sensory and efferent pathways as well as the neurochemical contributions involved. The authors demonstrate extensive expertise in this field, effectively synthesizing current knowledge. Overall, the review is well-structured and well-written, making it accessible to a broad readership.

However, certain aspects would benefit from further revision or more in-depth discussion to enhance clarity and completeness. Below, I outline my constructive comments and suggestions for improvement.

1) Page 3, line 24: While Ptgds is strongly expressed in the POA and is involved in thermoregulation, it is not a specific genetic marker for temperature-sensitive neurons since it is not uniquely restricted to temperature-sensitive neurons. This should be considered and reported in the review.

2) Page 3, lines 46-49: The authors stated that "chemokine receptor 4 (Ccr4) which binds CC Chemokine ligand 22 (Ccl22) to induce hyperthermia via activation of the brown adipose tissue (Osborn et al., 2011)". Is it hyperthermia or fever? On the same subject, Line 60, page 9; the authors reported that chemo- photogenic activation of QRFP mice led to hypothermia. Is this a regulated hypothermia? It would be good to differentiate fever from hyperthermia and hypothermia from regulated hypothermia (anapyrexia).

3) Page 4, lines 19-22: The authors stated that "Cold activates glutamatergic PoA neurons whereas warm activates GABAergic PoA neurons which excite or inhibit the sympathetic premotor command neurons to increase or reduce, respectively, the sympathetic drive." However, during heat exposure, sympathetic activity is also increased to promote sweating (McAllen, R. M., & McKinley, M. J. (2018). Thermoregulation: From Basic Neuroscience to Clinical Neurology Part I, 305-316. doi:10.1016/b978-0-444-63912-7.00018-7). Therefore, this explanation should be expanded.

4) Page 9, line 16: As the authors may be aware, the term "set-point" has faced criticism and debate, as evidence suggests that thermoeffectors can be activated or inhibited independently during thermoregulatory responses (for review see Branco LG et al. 2014; Compr Physiol. Oct;4(4):1301-38. doi: 10.1002/cphy.c130053). Therefore, I suggest replacing "set point" to "adaptive reference mechanism".

5) Some questions that also should be discussed:

a) How does brown adipose tissue (BAT) activation via hypothalamic pathways impact food-seeking behavior?

b) Does the hypothalamic response to cold exposure override hunger signals, or do they operate

independently?

c) How does fever (or other forms of hyperthermia) alter hypothalamic control of appetite and energy expenditure?

d) Do tanycytes respond differently to changes in core body temperature versus environmental temperature?

e) What ion channels or receptors enable tanycytes to detect thermal fluctuations?

f) How do tanycytes respond to fever and illness-induced anorexia?

6) Regarding the section "The role of tanycytes in thermal regulation and food-intake"; there is a very interesting study showing seasonal changes in tanycytes morphology in the Arctic ground squirrel (Urocitellus parryii) (Frare and Drew, 2021 - J Chem Neuroanat. 113:101920. doi: 10.1016/j.jchemneu.2021.101920)

7) The resolution of the figures is very low.

8) Figure 1 is somewhat difficult to interpret and could benefit from improved clarity. A more effective approach would be to place the specific stimuli that activate the channels inside parentheses, making it immediately clear what triggers each channel. This recommendation also applies to Figures 3 and 4, where similar adjustments would enhance readability and comprehension. Additionally, while the figure provides a useful overview, it represents a highly simplified model. It is important to acknowledge that cold- and warm-sensitive neurons in the POA express a wide array of receptors beyond those depicted. Including a brief mention of this in the figure legend or the main text would provide a more accurate representation of the complexity of thermosensory processing.

9) Figure 2 it is not so didactic. It is actually difficult to follow. A very nice model is presented by Romanovsky et al. (2009) - Pharmacological Reviews 61:228-261; doi:10.1124/pr.109.001263 - (Figure 1).

Minor
Standardize the abbreviation for preotic area. In some parts it is POA and in others PoA
